# Detection of carbapenem-resistant Klebsiella pneumoniae strains harboring carbapenemase, beta-lactamase and quinolone resistance genes in intensive care unit patients

**DOI:** 10.3205/dgkh000366

**Published:** 2020-11-30

**Authors:** Ozge Unlu, Mehmet Demirci

**Affiliations:** 1 Beykent University School of Medicine, Department of Medical Microbiology, Istanbul, Turkey

**Keywords:** carbapenem-resistant Klebsiella pneumoniae, beta lactamase, carbapenemase, quinolone resistance

## Abstract

**Aim:** Carbapenem-resistant *Klebsiella pneumoniae* (CR-Kp) strains are important nosocomial pathogens worldwide. In this study, we aimed to reveal the antibiotic resistance of clinical CR-Kp strains and determine the presence of KPC, OXA-48, VIM and IMP carbapenemase genes. CTX-M-1, TEM-1, SHV-1 extended-spectrum beta-lactamase (ESBL) genes, *qnrA*, *qnrB*, *qnrS* plasmid-mediated quinolone resistance genes and sul1 and sul2 sulfonamide resistance genes provided molecular epidemiological data.

**Methods:** A total of 175 *K. pneumoniae* strains were isolated from clinical samples of patients hospitalised in an intensive care unit (ICU) betweent April and October 2017. The strains were identified with conventional methods, with VITEK 2 (BioMerieux, France) and MALDI-TOF MS (Bruker, USA). Antimicrobial susceptibilities were tested using the disc-diffusion method and E-test (BioMerieux, France). Antimicrobial resistance genes were investigated via real-time PCR in strains identified as CR-Kp.

**Results:** High frequencies of *bla_TEM-1_* (86.36%), *bla_SHV-1_* (86.36%), and *bla_CTX-M-1_* (95.45%) genes were found in CR-Kp strains. Morever, all three ESBL genes coexisted in 77.3% of all strains. *bla_KPC_* was detected in 12 (54.55%) of the strains, and 4 of them which had an MIC> 16 μg/mL to imipenem showed *bla_OXA-48_* positivity as well. The *qnrS* gene determinant (86.36%) had the highest frequency, and strains carrying *qnrA* showed higher MICs for ciprofloxacin.

**Conclusion:** CR-Kp strains are able to develop different antimicrobial resistance patterns according to regional changes in antimicrobial therapeutic policies. Thus, it is important to monitor the regional molecular epidemiological data for efficient treatment.

## Zusammenfassung

**Ziel:** Carbapenem-resistente *Klebsiella pneumoniae* (CR-Kp)-Stämme sind weltweit wichtige nosokomiale Krankheitserreger. In dieser Studie wollten wir die Antibiotikaresistenz klinischer CR-Kp-Stämme aufdecken und KPC-, OXA-48-, VIM- und IMP-Carbapenemase-Gene nachweisen. CTX-M-1, TEM-1, SHV-1 Beta-Lactamase-Gene (ESBL) mit erweitertem Spektrum, *qnrA-*, *qnrB-*, *qnrS*-Plasmid-vermittelte Chinolon-Resistenzgene und *sul1*- und *sul2*-Sulfonamid-Resistenzgene dienten der Bereitstellung molekularer epidemiologischer Daten.

**Methode:** Insgesamt 175 *K. pneumoniae*-Stämme wurden zwischen April und Oktober 2017 aus klinischen Proben von Intensivtherapiepatienten isoliert. Die Stämme wurden mit herkömmlichen Methoden, mit VITEK 2 (BioMerieux, Frankreich) und mit MALDI-TOF-MS identifiziert (Bruker, USA). Die Empfindlichkeit wurden im Plättchendiffusions- und E-Test (BioMerieux, Frankreich) bestimmt. Antimikrobielle Resistenzgene wurden mittels Echtzeit-PCR in Stämmen untersucht, die als CR-Kp identifiziert wurden.

**Ergebnisse:** Die Ergebnisse zeigten eine hohe Häufigkeit von *bla_TEM-1_*, *bla_SHV-1_* und *bla_CTX-M-1_*-Genen in CR-Kp-Stämmen mit 86,36%, 86,36% bzw. 95,45%. Darüber hinaus waren alle drei ESBL-Gene in 77,3% aller Stämme gleichzeitig vorhanden. *bla_KPC_* wurde in 12 (54,55%) der Stämme nachgewiesen. 4 von ihnen, die eine MHK von mehr als 16 μg/mL gegen Imipenem hatten, zeigten ebenfalls *bla_OXA-48_* Positivität. Die *qnrS*-Gendeterminante (86,36%) wurde als am höchsten befunden, und *qnrA* tragende Stämme zeigten höhere MHK-Werte für Ciprofloxacin.

**Schlussfolgerung:** CR-Kp-Stämme können je nach Antibiotikastrategie unterschiedliche Resistenzmuster entwickeln. Daher ist es wichtig, die regionalen molekularen epidemiologischen Daten für eine effiziente Behandlung zu überwachen.

## Introduction

Carbapenem-resistant *Klebsiella pneumoniae* (CR-Kp) strains are considered to be important nosocomial pathogens worldwide, which can cause serious infections with high morbidity and mortality rates [1]. In particular, during the last decade, the global rates of infection with CR-Kp strains have increased substantially, a cause for serious concern [2]. Gram-negative bacteria are known to gain carbapenem resistance by the production of metallo-β-lactamases (MBLs) or non-metallo-carbapenemases (such as *Klebsiella pneumoniae* carbapenemases [KPCs]). Resistance to carbapenems in *K. pneumoniae* is also associated with the production of particularly potent carbapenemases and many other mechanisms. Alterations in cell membrane and beta-lactamases that promote poor carbapenemase activity may also contribute to carbapenem resistance [3], [4]. CR-Kp isolates generally possess multidrug resistance, including resistance to penicillins, third-generation cephalosporins, fluoroquinolones, and aminoglycosides. Previous studies demonstrated co-propagation of carbapenemase and extended-spectrum beta-lactamases (ESBLs) [5]. The prevalences of multidrug resistant pathogens such as CR-Kp may vary according to region, country, and susceptible population. The severity of the problem is significantly related to the regional measures implemented to control the spread of resistant bacteria [6]. Since there is no antimicrobial that can be used to treat infections caused by newly defined pan-resistant CR-Kp strains, and because these strains are capable of developing resistance very rapidly to each new antibiotic developed, it is important to know their epidemiology in order to investigate alternative treatment options and analyze the virulence properties of this bacterium to prevent global spread [7]. The purpose of this study was to elucidate the antibiotic resistance patterns of CR-Kp strains detected as nosocomial pathogens, determine KPC, OXA-48, VIM and IMP carbapenemase gene frequencies and investigate the genes responsible for ESBL production commonly found in *K. pneumoniae* strains, such as CTX-M-1, TEM-1, and SHV-1. In addition, *qnrA*, *qnRB1*, *qnRS1* plasmid-mediated quinolone resistance determinants that are closely related to ESBL genes and responsible for low-level quinolone resistance were investigated, as were *sul1* and *sul2* sulfonamide resistance genes, to provide molecular epidemiological data about these strains.

## Materials and Methods

### Bacterial strains

A total of 175 *Klebsiella pneumoniae* strains isolated from blood samples of ICU patients admitted to a university hospital in Turkey between April and October 2017 were evaluated. In all cultures, pathogens were identified and their antimicrobial susceptibilities determined by conventional laboratory methods and confirmed by the VITEK-2 system (BioMerieux, France). Isolate identification was confirmed with matrix-assisted laser desorption ionization time-of-flight mass spectrometry (MALDI-TOF-MS; Bruker, USA). The antimicrobial susceptibilities of these strains were determined using the Kirby-Bauer disc. diffusion method, but colistin and the MICs for ciprofloxacin and imipenem were determined using the E-test (BioMerieux, France) on Mueller-Hinton agar media. Colistin susceptibility was determined by the standard microdilution method. Susceptibility results were interpreted according to the EUCAST clinical breakpoints [8]. CR-Kp was defined as an isolate with imipenem MIC>2 µg/mL. According to MIC results, non-duplicate *Klebsiella pneumoniae* (n=22) strains were identified as CR-Kp and were included in this study.

### DNA extraction

A single colony of each strain’s overnight culture on Eosin methylene blue (EMB) agar was suspended in 50 mL of ultrapure water. The suspension was heated at 95°C for 10 min and centrifuged at 14,000 rpm for 10 min. Thirty μL of the supernatant were used as a DNA template for real-time PCR [9].

### Antimicrobial resistance genes

CR-Kp strains were analyzed for the presence of *bla_KPC_*, *bla_OXA-48_*, *bla_VIM_*, and *bla_IMP_* carbapenem resistance genes, *bla_TEM-1_*, *bla_SHV-1_*, and *bla_CTX-M-1_* extended-spectrum beta-lactamases genes, and *qnrA*, *qnrB*, and *qnrS* plasmid-mediated quinolone resistance genes using real-time PCR. Because sulfonamide is a common therapeutic alternative in clinics to beta lactam antibiotics, resistance to trimethoprim–sulfamethoxazole was also assayed by examining *sul1* and *sul2* sulfonamide resistance genes. Table 1 (Tab. 1) demonstrates sequences of these primers [9]. 

The *LightMix Modular Assay* (Roche Diagnostics GmbH, Mannheim, Germany) was utilized to detect *bla_KPC_*, *bla_OX__A-4__8_*, *bla_VIM_*, and *bla_IMP_* genes with multiplex real-time PCR with the LightCycler 480 II system (Roche Diagnostics GmbH, Mannheim, Germany) according to the manufacturer’s instructions. Specific primers and the LightCycler 480 Sybr Green I Master kit was used in the LightCycler 480 II system (Roche Diagnostics GmbH, Mannheim, Germany) to detect *bla_TEM-1_*, *bla_SHV-1_*, and *bla_CTX-M-1_*, *qnrA1*, *qnrB1*, *qnrS1*, *sul1*, and *sul2* genes. The real-time PCR run-profile was as follows: denaturation at 95°C for 10 min, followed by 45 cycles of 10 s at 95°C, 30 s at 55°C, and 1 s at 72°C.

## Results

In this study, we evaluated 22 CR-Kp strains out of 175 *K. pneumoniae* strains. The mean (±SD) age of the patients in whom CR-Kp strains were isolated was 39.41±17.47. According to Kirby-Bauers’s disc-diffusion tests, all strains were resistant to all investigated penicillins and cephalosporins, including 3^rd^ generation cephalosporins and carbapenems (Table 2 (Tab. 2)).

Analysis of Imipenem and Ciprofloxacin MICs in CR-Kp strains revealed that the MIC50 and MIC90 values for imipenem were 8 and 32 μg/mL, respectively, and for ciprofloxacin the MIC50 and MIC90 values were 8 and 64 μg/mL, respectively (Table 3 (Tab. 3)). Real-time PCR results revealed high frequencies of *bla_TEM-1_*, *bla_SHV-1_*, and *bla_CTX-M-1_* genes in CR-Kp strains, i.e., 86.36%, 86.36%, and 95.45%, respectively. Morever, 77.3% of all strains carried three ESBL genes together (Table 4 (Tab. 4)). 

When the beta-lactamase and carbapenemase gene determinants detected in 22 CR-Kp strains were examined together, *bla_CTX-M-1_* positivity was found to be the highest (95.45%) compared to other genes. Different patterns were detected in the resistance analysis of 22 CR-Kp strains. Eight strains carried CTX-M-1+TEM+SHV, 5 strains carried CTX-M-1+TEM+SHV+KPC, and 4 strains CTX-M-1+TEM+SHV+KPC+OXA-48 together (Table 5 (Tab. 5)). *bla*_KPC_ was detected in 12 (54.55%) of the strains and 4 of them – which had an MIC>16 μg/mL to imipenem according to the E-test – showed *bla*_OXA-48_ positivity as well. Neither *bla*_IMP_ nor *bla*_VIM_ were detected in any of the strains. Also, all strains were found positive for the *sul1* gene and one strain was found negative for the *sul2* gene. 

Moreover three strain (5, 9, 11) that were resistant to all tested antimicrobials (except colistin) according to Kirby Bauer’s disc-diffusion test were found positive to all *bla_CTX-M-1_*, *bla_SHV-1_*, *bla*_TEM-1_, *qnrB*, *qnrS*, *sul1*, *sul2*, *bla*_KPC_ and *bla*_OXA-48_ genes.

Although all strains resistant to ciprofloxacin carried at least one plasmid-mediated quinolone resistance gene, the frequencies of the genes responsible for the resistance were found to be different among the strains. While the *qnrS* gene determinant had the highest frequency (86.36%), the *qnrA* gene determinant showed a relatively low frequency (27.27%) (Table 6 (Tab. 6)).

## Discussion

Over the last decades, *Klebsiella pneumoniae* (KP) and carbapenem-resistant *K. pneumoniae* (CR-Kp) have emerged as a global public health problem, leading to community-acquired invasive infections. Especially CR-Kp strains producing different beta-lactamases are now very common in the community and resistant to frequently used antimicrobial options [10], [11]. Accurate and early identification of CR-Kp and revealing its beta-lactamases and carbapanemases are important for efficient treatment and to control pandemic CR-Kp infections [12], [13]. As the incidence of CR-Kp strains and drug resistance profiles can vary according to region, country and sensitive population, it is important to have epidemiological data [6]. Given the fact that the first case of OXA-48 originating from CR-Kp was reported from Turkey, the importance of monitoring the molecular epidemiological profile in our country is apparent [14].

In previous studies reporting the antimicrobial resistance profiles of CR-Kp strains, Zhang et al. found that the resistance rates of 41 CR-Kp strains to antimicrobials were 24.4% for amikacin, 100% for cephazidime, 26.8% for ciprofloxacin, 43.9% for gentamicin and 36.6% for trimethoprim sulfamethoxazole [4]. Ocampo et al. reported antimicrobial resistance of 193 CR-Kp strains as 76% for ciprofloxacin, 49.7% for gentamicin, and 36.3% for amikacin [15]. In 2015, Zhang et al. reported that the CR-Kp strain isolated from 5 patients showed 80% resistance to amikasin, 100% to ciprofloxacin and 20% to tigecycline. Also, the MIC90 values for ciprofloxacin and imipenem of CR-Kp strains were 64 and 32, respectively, similar to our study [16]. Zheng et al. reported the resistance rate as 100% for ceftazidime, imipenem, and ciprofloxacin, 95% for amikacin, 16% for trimethoprim/sulfamethoxazole, and 0% for tigecycline in a study conducted with 100 CR-Kp strains. Those authors also found MIC90 >128 for imipenem [17]. Yan et al. reported the resistance rates as 59% for ciprofloxacin and 47.4% for gentamicin in a study conducted in 2017 with 78 CR-Kp strains [18]. In a different study, Zhang et al. reported a resistance rate of 24.4% for amikacin, 26.8% for ciprofloxacin, 36.6% for trimethoprim-sulfamethoxazole, and 43.9% for gentamycin for 41 CR-Kp strains [4]. Neuner et al. found the resistance of 60 CR-Kp strains to be 2% for tigecycline, 55% for amikacin and 78% for gentamicin [19]. In a study conducted with 37 CR-Kp strains in Turkey, Iraz et al. reported 100% resistance to ceftazidime, imipenem, and meropenem, 2.7% to colistin, 11% to tigecycline, 19% to amikacin, and 21.6% to trimethoprim/sulfamethoxazole [20]. In another study conducted in Turkey, Candevir et al. reported 12.5% tigecycline, 72% amikacin and 90% ciprofloxacin resistance in CR-Kp strains [21]. Taking all these studies into consideration, it can be seen that differences exist in the antibiotic resistance profiles of CR-Kp strains even from region to region, which suggests that CR-Kp strains are influenced by the regional treatment policies and may produce different resistance profiles against antibiotics. 

According to the previous studies conducted with CR-Kp strains and their beta lactamases, Ocampo et al. reported finding SHV in 100% of strains, KPC in 86%, TEM in 45% and VIM gene in only 1 strain, whereas OXA-48 and IMP genes were not detected in any of the strains [14]. Iraz et al., in their study conducted with 37 CR-Kp strains, detected OXA-48 in 32 (86%) strains, CTX-M-1 in 23 (62%), SHV in 31 (97%) and TEM in 29 (92%) strains, but none of the strains were found to encode IMP and VIM genes [20]. Satlin et al. found that 97 CR-Kp strains carried 94%, 85%, 64%, 8% and 1% KPC, TEM, SHV, CTX and OXA-48, respectively [22]. Zhang et al. reported that the rate of coexistence of KPC+TEM+SHV in CR-Kp strains was the most frequently observed, followed by KPC+TEM [23]. In another study, Zhang et al. found CTX-M+TEM+SHV coexistence in all of the CR-Kp strains isolated from 5 patients [16]. In our study,

- CTX-M-1+TEM+SHV coexistence was found in 8,
- CTX-M-1+TEM+SHV+KPC in 5, and
- CTX-M-1+TEM+SHV+KPC+OXA-48 in 4 of the 22 CR-Kp strains.

Zheng et al. detected 75% SHV and 1% TEM among 100 CR-Kp strains [17]. In the same study, they also reported that CTX-M-1, OXA-48, VIM, and IMP were not detected in any of the strains. Furthermore, they found 92.7% positivity for SHV, 68.3% for TEM and 26.8% for IMP in their study conducted with 41 CR-Kp strains [4]. Also, it has been emphasized that coexistence of different gene determinants for the same antimicrobial group were frequently found in multi-drug resistant strains; hence, these strains were resistant to many antibiotics via many different pathways [4]. Similarly, we observed that different gene determinants coexiested in our clinical strains, phenotypically showing different antibiotic resistance patterns. Examining 78 CR-Kp strains, Yan et al. found 8 (10.3%) were positive for KPC, 65 (83.3%) for SHV, 28 (35.9%) for TEM, 46 (59%) for CTX-M-1, and 3 (3.8%) for IMP. In the same study, it was also reported that KPC-positive strains showed higher carbapenem MICs [18]. When this phenomenon was examined in our study, higher MICs were not detected in all of the KPC-positive strains. Considering the previous studies conducted in Turkey on beta-lactamase and carbapenemase genes in CR-Kp strains, Candevir et al. reported a positive rate of 74.5% for *bla_OXA-48_*, 67.3% for *bla_SHV-1_*, 60.2% for *bla_TEM-1_* and 45.9% for *bla_VIM_* in the CR-Kp strains [21]. As can be seen from the results of different studies conducted in China, there may be regional differences in the genes that the strains carry, in response to the regionally different policies of antibiotic usage. This suggests that the strain can activate the relevant gene as needed in the antimicrobial resistance process. Akya et al. reported that *K. pneumoniae* strains carrying *qnrB* have MICs from 32 to 128 μg/ml for Ciprofloxacin (except one strain which has an MIC between 4 and 16 μg/ml). Those authors also noticed the strains that carried *qnrB* and *qnrS* together had even higher MICs (≥256 μg/ml) for ciprofloxacin [24]. Our findings for *qnrB* differed from those of the study by Akya et al. However, it was clear that strains carrying *qnrA* has a higher MIC [24]. Zheng et al. reported a positivity rate of 8% for *qnrB*, and 4% for *qnrA* and *qnrS* in their study on 100 ciprofloxacin-resistant CR-Kp strains [23]. Yan et al. reported *qnrB* and *qnrS* positivity as 56.4% and 38.5%, respectively, in their study on 78 CR-Kp strains [18]. Zhang et al. found that 19.5% of the strains were *qnrS* and 14.6% were *qnrB* positive in their study performed with 41 CR-Kp strains [4]. However, *qnrA* was not found in any of the strains. Szabó et al. [25] detected *qnr* determinants in 8% of *K. pneumoniae* strains. In the same study, it was also reported that the MIC for Ciprofloxacin increased 132-fold in *qnrA*-positive strains. The relationship of *qnrA* to beta lactamase genes has also been examined, and *qnrA* was found to be associated with SHV [25]. Similar to this phenomenon in our study, 6 strains were detected as *qnrA* positive, and except for one strain (strain number: 21), the MICs for ciprofloxacin were 64 and above, and carried the SHV determinant. Despite its low frequency among the strains, *qnrA* positivity has a significant effect on the MICs of ciprofloxacin. According to previous studies on *qnr* gene determinants, similar to changes in antimicrobial resistance profiles, frequencies of *qnr* gene determinants among the strains vary from country to country, region to region and even hospital to hospital within the same region. This supports our earlier view, suggesting that CR-Kp strains are able to develop defense mechanisms, such as obtaining genes responsible from antimicrobial resistance via horizontal gene transfer, against different challenges in different regions to survive in the host.

## Conclusions

The results of our study demonstrated different gene determinants for beta-lactamase and carbapenemase activities at the same time in CR-Kp strains isolated from clinical specimens. In particular, CTX-M, SHV and TEM coexisted in many strains, and in *qnrA* positive strains, the MICs for ciprofloxacin were significantly higher. Thus, it is important to monitor the molecular epidemiologic regional data in order to succesfully treat infections caused by CR-Kp strains which may develop different antimicrobial resistance patterns according to regional variations in antimicrobial therapeutic policies. 

## Notes

### Competing interests

The authors declare that they have no competing interests.

### Author Contributions

UO, DM conceived, designed and performed the experiments, analysed the data and wrote the paper.

## Erratum

Correction of the title

## Figures and Tables

**Table 1:**
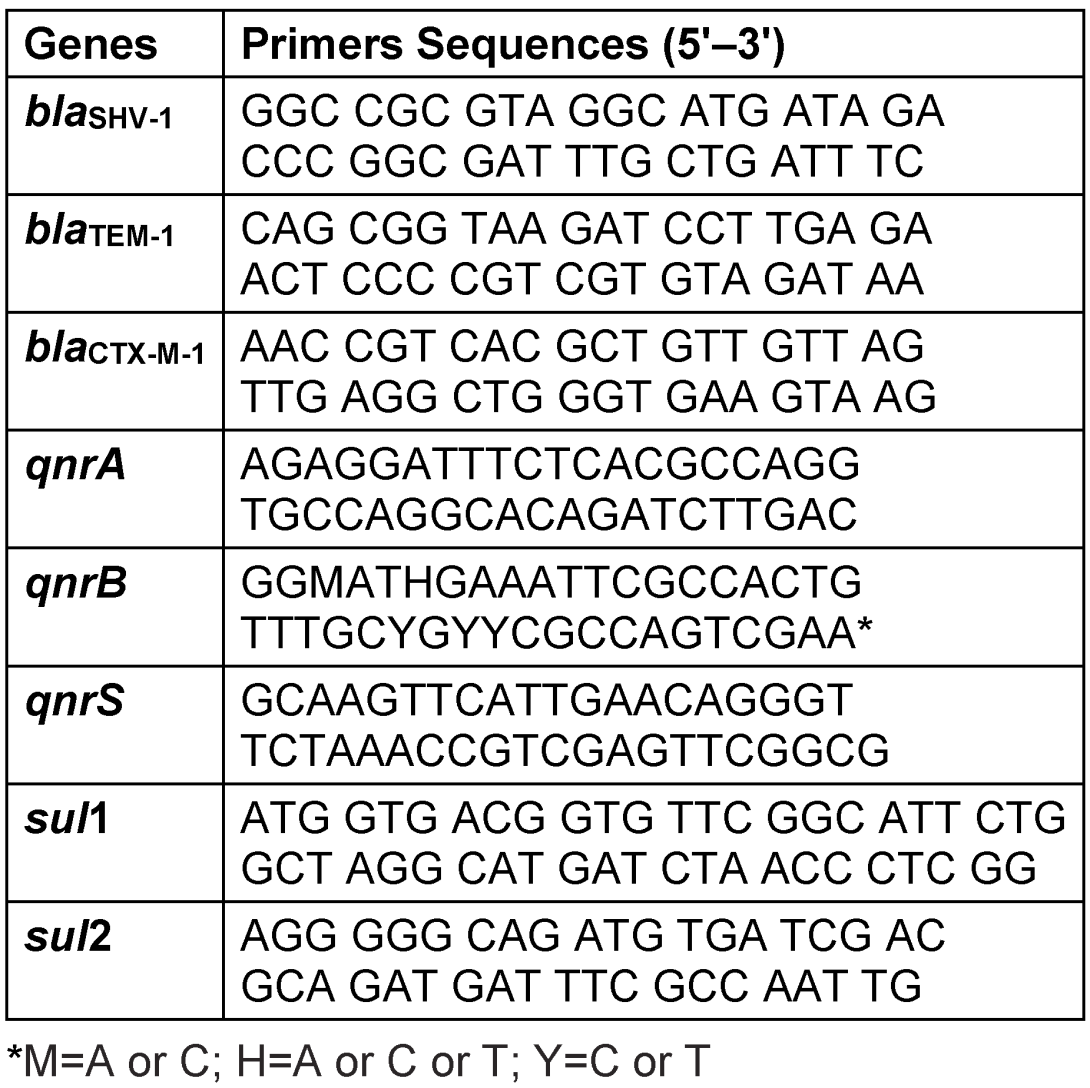
Primers related to antimicrobial resistance genes used in this study

**Table 2:**
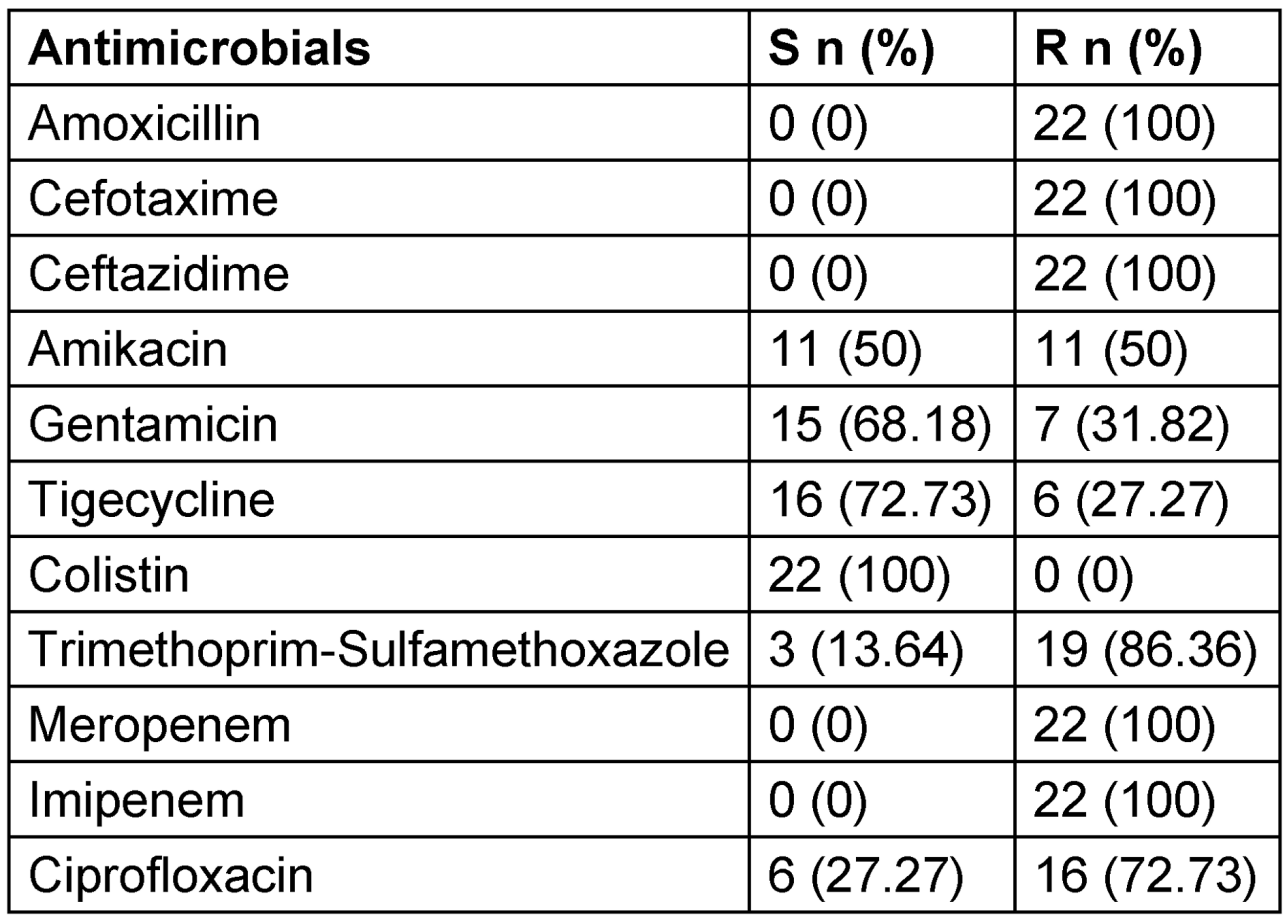
Antimicrobial susceptibility test results of CR-Kp strains

**Table 3:**
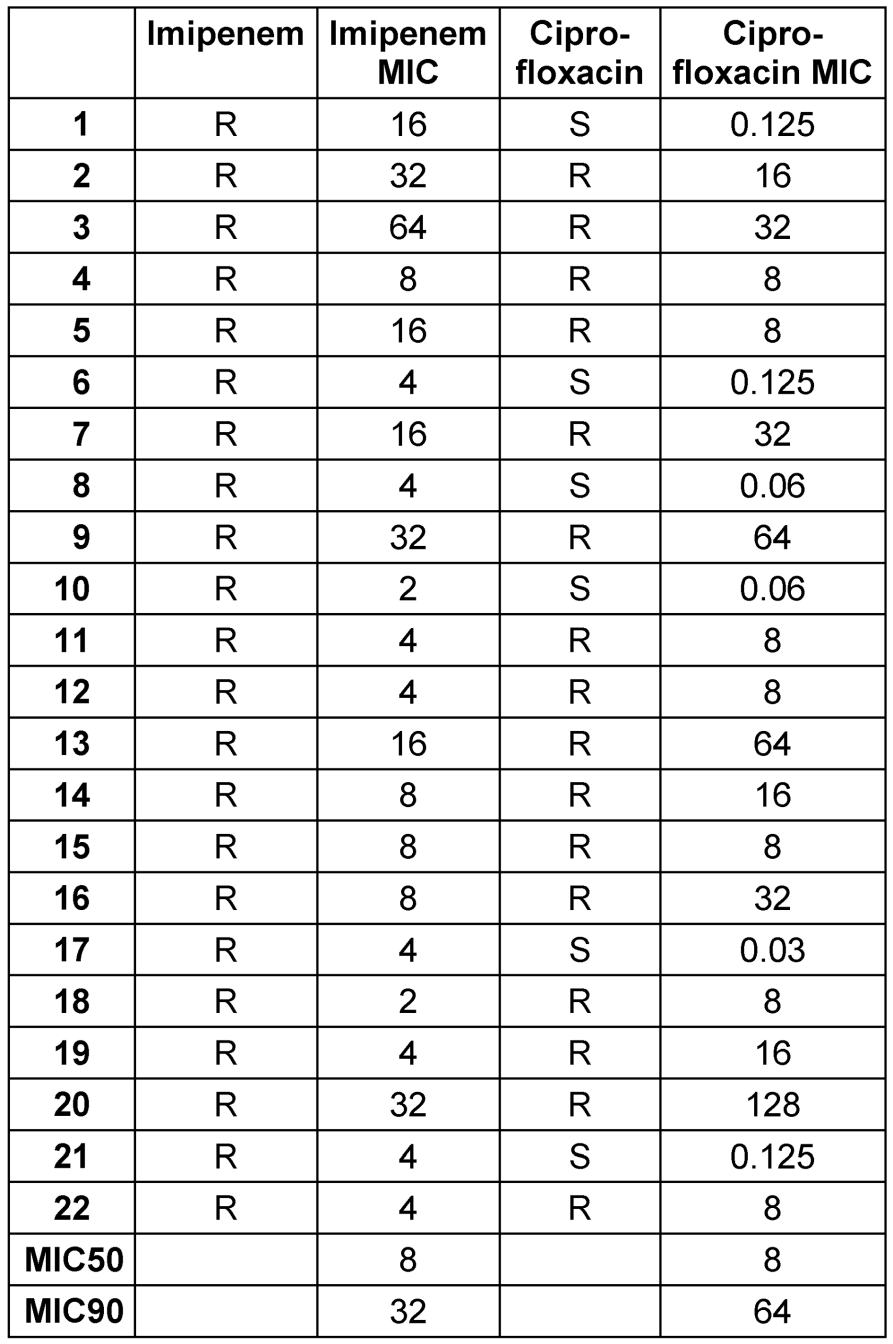
Imipenem and Ciprofloxacin MICs (μg/mL) against CR-Kp in this study

**Table 4:**
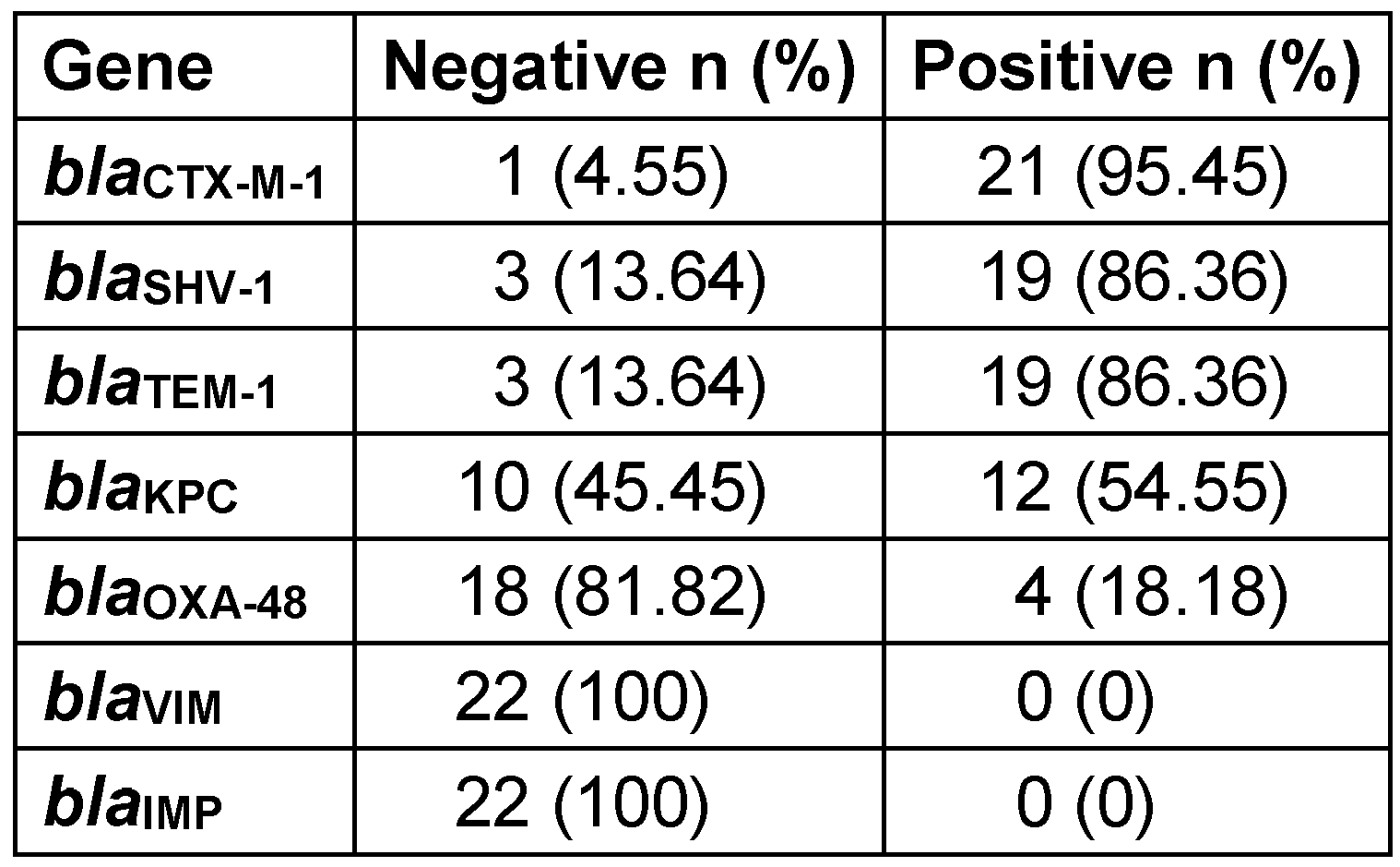
Distribution rates of beta-lactamase and carbapenemase genes in CR-Kp strains

**Table 5:**
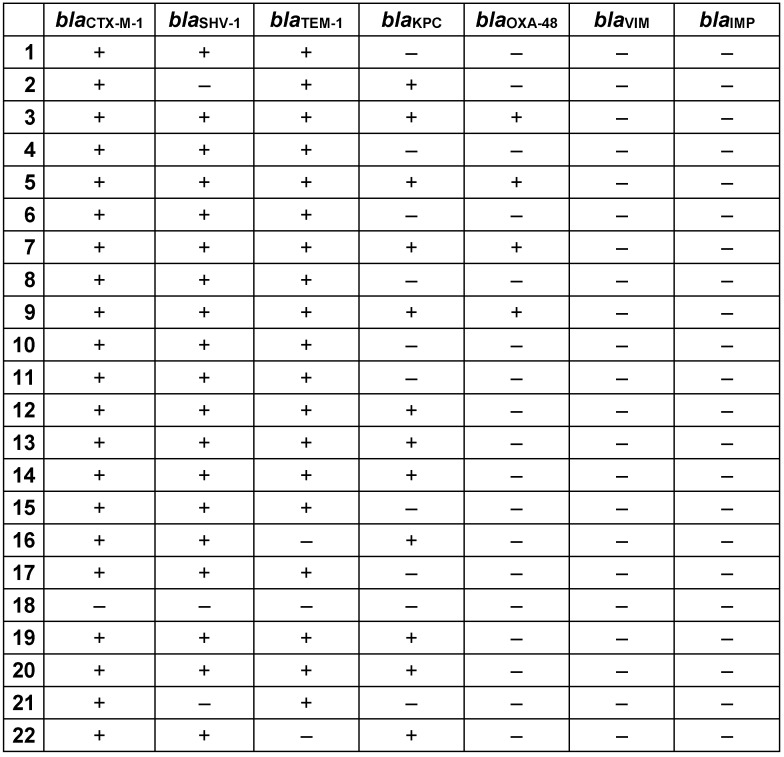
Distribution patterns of beta-lactamase and carbapenemase genes in CR-Kp strains

**Table 6:**
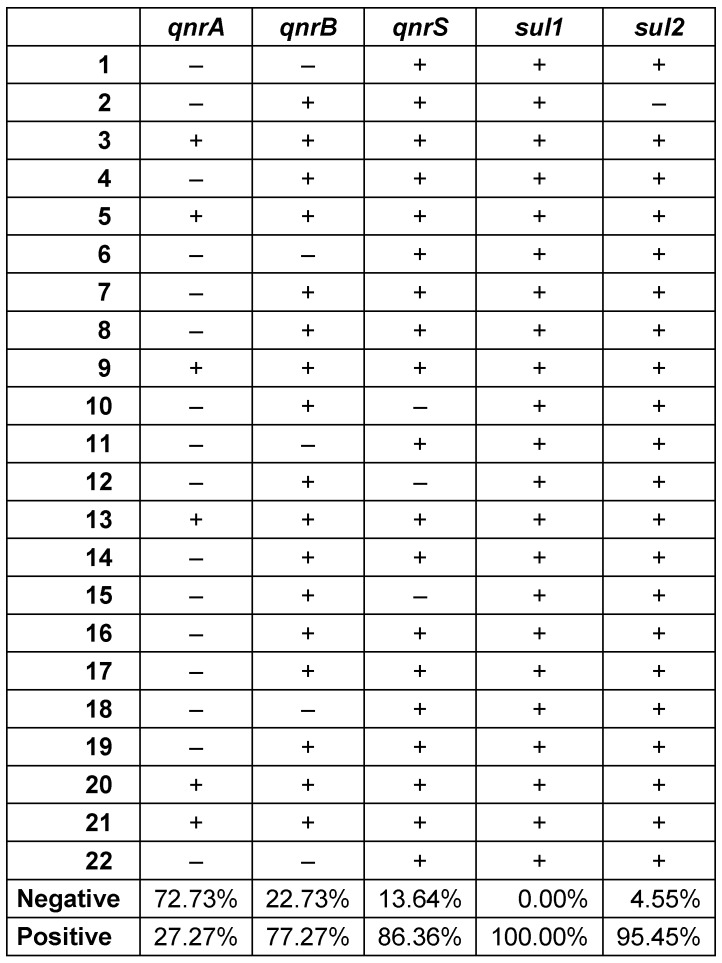
Distribution of *qnr* and *sul* gene determinants in CR-Kp strains
